# Feasibility of Hypotension Prediction Index-Guided Monitoring for Epidural Labor Analgesia: A Randomized Controlled Trial

**DOI:** 10.3390/jcm14145037

**Published:** 2025-07-16

**Authors:** Okechukwu Aloziem, Hsing-Hua Sylvia Lin, Kourtney Kelly, Alexandra Nicholas, Ryan C. Romeo, C. Tyler Smith, Ximiao Yu, Grace Lim

**Affiliations:** 1 Department of Anesthesiology and Perioperative Medicine, University of Pittsburgh School of Medicine, Pittsburgh, PA 15224, USA; 2 Department of Obstetrics, Gynecology & Reproductive Sciences, University of Pittsburgh School of Medicine, Pittsburgh, PA 15224, USA; 3 UPMC Magee, 300 Halket Street, Suite 3403, Pittsburgh, PA 15213, USA

**Keywords:** epidural, childbirth, hypotension, prediction, maternal hemodynamics, vasopressor, obstetric

## Abstract

**Background:** Hypotension following epidural labor analgesia (ELA) is its most common complication, affecting approximately 20% of patients and posing risks to both maternal and fetal health. As digital tools and predictive analytics increasingly shape perioperative and obstetric anesthesia practices, real-world implementation data are needed to guide their integration into clinical care. Current monitoring practices rely on intermittent non-invasive blood pressure (NIBP) measurements, which may delay recognition and treatment of hypotension. The Hypotension Prediction Index (HPI) algorithm uses continuous arterial waveform monitoring to predict hypotension for potentially earlier intervention. This clinical trial evaluated the feasibility, acceptability, and efficacy of continuous HPI-guided treatment in reducing time-to-treatment for ELA-associated hypotension and improving maternal hemodynamics. **Methods:** This was a prospective randomized controlled trial design involving healthy pregnant individuals receiving ELA. Participants were randomized into two groups: Group CM (conventional monitoring with NIBP) and Group HPI (continuous noninvasive blood pressure monitoring). In Group HPI, hypotension treatment was guided by HPI output; in Group CM, treatment was based on NIBP readings. Feasibility, appropriateness, and acceptability outcomes were assessed among subjects and their bedside nurse using the Acceptability of Intervention Measure (AIM), Intervention Appropriateness Measure (IAM), and Feasibility of Intervention Measure (FIM) instruments. The primary efficacy outcome was time-to-treatment of hypotension, defined as the duration between onset of hypotension and administration of a vasopressor or fluid therapy. This outcome was chosen to evaluate the clinical responsiveness enabled by HPI monitoring. Hypotension is defined as a mean arterial pressure (MAP) < 65 mmHg for more than 1 min in Group CM and an HPI threshold < 75 for more than 1 min in Group HPI. Secondary outcomes included total time in hypotension, vasopressor doses, and hemodynamic parameters. **Results:** There were 30 patients (Group HPI, n = 16; Group CM, n = 14) included in the final analysis. Subjects and clinicians alike rated the acceptability, appropriateness, and feasibility of the continuous monitoring device highly, with median scores ≥ 4 across all domains, indicating favorable perceptions of the intervention. The cumulative probability of time-to-treatment of hypotension was lower by 75 min after ELA initiation in Group HPI (65%) than Group CM (71%), although this difference was not statistically significant (log-rank *p* = 0.66). Mixed models indicated trends that Group HPI had higher cardiac output (β = 0.58, 95% confidence interval −0.18 to 1.34, *p* = 0.13) and lower systemic vascular resistance (β = −97.22, 95% confidence interval −200.84 to 6.40, *p* = 0.07) throughout the monitoring period. No differences were found in total vasopressor use or intravenous fluid administration. **Conclusions:** Continuous monitoring and precision hypotension treatment is feasible, appropriate, and acceptable to both patients and clinicians in a labor and delivery setting. These hypothesis-generating results support that HPI-guided treatment may be associated with hemodynamic trends that warrant further investigation to determine definitive efficacy in labor analgesia contexts.

## 1. Introduction

Despite decades of safe use, hypotension after epidural labor analgesia (ELA) remains the most common complication and is experienced by about one of every five patients receiving ELA. Hypotension has safety ramifications to both mother and fetus and results in, at its mildest, maternal lightheadedness and nausea and, at its most severe, fetal acidemia and difficulties with fetal-to-neonatal transition. These safety concerns make early detection, prevention, and treatment of ELA-associated hypotension a critical component for patient safety on labor and delivery units worldwide.

As digital technologies transform perioperative medicine, there is growing interest in leveraging predictive analytics and real-time physiologic monitoring to support clinical decision-making. Tools like the Hypotension Prediction Index (HPI) exemplify this shift, offering clinicians early warning of hemodynamic instability based on continuous arterial waveform analysis. In dynamic labor and delivery environments, where maternal physiology evolves rapidly and treatment windows are narrow, such digital decision support systems may facilitate earlier, more targeted interventions to protect both maternal and fetal well-being.

Currently, conventional monitoring involves intermittent non-invasive blood pressure checks to detect and treat hypotension. The current American Society of Anesthesiologists Guidelines on Obstetric Anesthesia Practice [1] recommend monitoring for hypotension, with most practices using non-invasive cuff measures, typically expected every 5–15 min. However, intermittent monitoring may result in delays in recognition and treatment of low blood pressure [2]. Delays in treating epidural anesthesia-associated hypotension can result in inadequate maternal–fetal perfusion [2]. Further, causes of hypotension after neuraxial in obstetric patients are primarily driven by reductions in systemic vascular resistance (SVR) [3]. Early detection of ELA-associated hypotension is critically needed to facilitate earlier treatments with vasopressors, typically consisting of either ephedrine or phenylephrine, to prevent hypotension-related morbidities. Further, tailored treatment to maternal hemodynamics—a tactic made possible using HPI—is important because changes in systemic vascular resistance (SVR) and cardiac output (CO) during labor are known to impede uterine blood flow and fetal oxygenation. These changes can lead to fetal acidemia and distress, which can then lead to preventable and emergent birth interventions.

Our proposed solution involves using the Hypotension Prediction Index (HPI) (ClearSight^TM^, Edwards Lifesciences, Irvine, CA, USA) to reduce time-to-treatment of epidural labor analgesia (ELA)-associated hypotension and to improve maternal hemodynamics, resulting in improved fetal perfusion. The HPI algorithm on ClearSight^TM^ is a machine learning-based decision support tool that analyzes arterial waveform features to predict hypotension, defined by mean arterial pressure (MAP) < 65 mmHg for at least 1 min [4]. The index ranges 0 to 100, with higher numbers reflecting a higher likelihood of subsequent hypotension. HPI has 92% sensitivity and specificity for predicting hypotension 5 min in advance, sensitivity 89% and specificity 90% for 10 min in advance, and 88% and 87% for 15 min in advance [4]. The sensitivity and specificity of HPI to accurately predict hypotension have also been validated with conventional invasive and non-invasive arterial waveforms, in both pregnant and non-pregnant cohorts [4,5,6,7,8,9]. Other studies have similarly demonstrated the effectiveness of HPI in predicting hypotension in perioperative settings [10,11,12]. The algorithm is FDA-approved for sale in Europe and the United States. To our knowledge, no study to date has rigorously assessed the utility of HPI in ELA compared to conventional monitoring, for the benefits of reduced time-to-treatment of ELA-associated hypotension.

Although prior studies have evaluated the use of ClearSight™ and HPI in obstetric settings and primarily under cesarean delivery, none have specifically focused on implementation-specific (patient- and clinician-specific) outcomes in a vaginal birth context under neuraxial analgesia. This study was designed to assess real-world workflow integration, to evaluate clinician and patient perceptions of the monitoring approach, and to generate preliminary estimates of treatment effect size to inform future research.

As healthcare systems embrace predictive algorithms and automated monitoring, integrating these tools into dynamic labor workflows requires evidence not only of efficacy, but of real-world feasibility and user acceptance. The purpose of this randomized controlled trial was primarily to evaluate the feasibility, appropriateness, acceptability, and preliminary efficacy of HPI-guided management compared to conventional monitoring. We accomplished this objective by assessing a randomized controlled trial protocol designed to assess time-to-treatment of hypotension in healthy pregnant individuals receiving epidural labor analgesia (ELA). We hypothesized that HPI-guided monitoring during labor epidural analgesia would be feasible and acceptable to patients and clinicians.

## 2. Methods

### 2.1. Study Design and Population

This was a prospective single-center randomized controlled trial designed to assess feasibility, acceptability, and preliminary efficacy (Clinical Trials.Gov information: NCT05906368, registered 15 June 2023). All methods were carried out in accordance with relevant guidelines and regulations, and all protocols were approved by the University of Pittsburgh Institutional Review Board (STUDY23030009). Written informed consent was obtained from subjects. Subjects were eligible if they were admitted to the hospital for labor and delivery, pregnant at term gestation, and receiving epidural labor analgesia (ELA). Exclusion criteria included non-reassuring fetal tracing at the time of ELA request, unintentional dural puncture, contraindications to ELA, significant cardiac arrhythmias or aortic regurgitation, arrhythmia, treatment with antihypertensive medications, pre-eclampsia with or without severe features, preoperative infection, inability to use the study device for any reason, and incomplete data.

### 2.2. Randomization and Interventions

After informed consent, enrolled subjects were randomized (1:1) to treatment of hypotension according to conventional monitoring (Group CM) or monitoring by HPI (Group HPI). The total monitoring time was 4 h, starting from the initiation of ELA defined by the time of first dose of epidural medications. To enable group comparisons of total time in hypotension and other hemodynamic parameters, all participants wore continuous, non-invasive blood pressure monitoring (CBPM): Group CM was blinded to CBPM output, while Group HPI received treatment of hypotension according to CBPM output and as specified by the hypotension treatment protocol below.

In Group HPI, we used an index threshold for treatment that built upon findings from Maheshwari et al. [13]. In their trial of 214 non-cardiac surgical patients, 105 (49%) patients were randomized to management with a hypotension prediction algorithm, and intraoperative hypotension was not reduced compared with controls. They suggested that a lower alert threshold, enabling adequate warning time and a simpler treatment algorithm that emphasizes prompt treatment after alert, may be useful. Therefore, our HPI alert threshold for treatment was specified at 75, and the treatment algorithm, specified below, was made as simple as possible.

### 2.3. Pre-ELA Protocol

Subjects received a 500 mL crystalloid co-load that started during placement of ELA. Pregnant women in the lateral decubitus position can measure 10 mmHg differences in dependent and upper arm blood pressures [14]. Therefore, prior to ELA initiation, the anticipated initial post-ELA decubitus position of the patient was clarified as right or left lateral decubitus. Standard monitoring was applied, including pulse oximeter and non-invasive blood pressure (NIBP) cuff affixed over the non-dependent arm. In Group HPI, both CBPM and NIBP monitors were applied on the same arm.

### 2.4. ELA Protocol

Baseline blood pressure was recorded immediately prior to the initiation of ELA. At subject request, a licensed and qualified anesthesia provider performed ELA using a combined spinal epidural (CSE) technique in the sitting position. CSE was chosen due to reliability and rapidity with which hypotension can be predicted within the ELA encounter. A meta-analysis of studies comparing low-dose epidural analgesia with CSE analgesia found no difference in the incidence of hypotension between the two techniques [15].

The epidural space was found using loss of resistance to saline. A 25 g or 27 g Sprotte needle was introduced to the subarachnoid space. After confirmation of cerebrospinal fluid (CSF), a spinal dose of fentanyl 15 mcg and bupivacaine 2.5 mg was given (this action defined T0 = initiation of CSE). At T0, blood pressure monitoring and data recordings began per the protocol specified below. A 19–20 g flexible epidural catheter was then introduced and threaded 5–6 cm into the epidural space. A test dose was delivered through the epidural catheter and consisted of 3 mL lidocaine 1.5% with epinephrine. The anesthesia clinician declared positive or negative test dose 5 min after administration of test dose. A positive test dose (defined as heart rate increase > 10 beats per minute at 30 s after injection, observation of both a metallic taste and tinnitus after injection, or warm or heavy sensation in the lower extremities at 3 min, or inability to raise legs within 4–10 min after injection) resulted in withdrawal from the study.

Maintenance of ELA was by patient-controlled epidural analgesia using programmed intermittent epidural bolus (PIEB) at our institutional standards: Ropivacaine 0.1% with fentanyl 2 mcg/mL, 8 mL every 40 min, 8 mL demand every 8 min, maximum hourly volume 24 mL (Smiths CADD^®^-Solis Infusion System, Infusion Pump, ICU Medical, Dublin, OH, USA). The first PIEB dose was delivered 40 min after the initiation of the pump.

### 2.5. Hypotension Definition

We defined hypotension as a mean arterial pressure (MAP) < 65 mmHg for more than 1 min. In Group CM, hypotension was measured by conventional non-invasive blood pressure cuff. In Group HPI, hypotension was measured by CBPM and alerted when HPI threshold was ≥75 for more than 1 min. Our hospital has existing protocols for epidural-associated hypotension to guide BP treatment: drop in systolic blood pressure (SBP) > 20% from baseline, or systolic blood pressure < 100 mmHg, or if there are any fetal heart rate concerns. These parameters continued per existing clinical standards if they were measured and detected by the NIBP regardless of group assignment and were expected to be evenly randomly distributed between the two groups by virtue of randomization. Random distribution of these events by frequency between groups was assessed in analysis. Hypotension treatment protocols were standardized as below.

### 2.6. Monitoring and Hypotension Treatment Protocols

Monitoring occurred as follows. In Group CM at the time of test dose delivery, automated NIBP cycles began and were set to read every 3 min for 30 min, amounting to a total of at least 10 NIBP measurements. After 30 min, NIBP was cycled to every 15 min by existing clinical standards. At 4 h, monitoring for the protocol ended. In Group HPI, at the time of test dose delivery, CBPM monitor continued. At 4 h, monitoring for the protocol ended.

*Hypotension Treatment Protocol: Group CM.* In Group CM, at the first hypotensive episode, a 500 mL intravenous fluid bolus was given, as well as ephedrine 10 mg intravenous push. At the second hypotensive episode, an ephedrine 10 mg intravenous push was given. For the third or more hypotensive episode, treatment was per physician anesthesiologist discretion. Ephedrine was selected as the first-line vasopressor in accordance with our institutional standards for treating neuraxial hypotension during labor, particularly given its beta-agonist profile favorable for maintaining maternal heart rate and cardiac output.

*Hypotension Treatment Protocol: Group HPI.* For Group HPI, the hypotension treatment protocol was tailored to individual hemodynamic parameters. In Group HPI, at the first HPI alert, the hemodynamic relations screen was consulted, and if the identified issue was one of preload, a 500 mL intravenous fluid bolus was given. Although SVV was available as part of the hemodynamic display, fluid responsiveness was not formally assessed using CO augmentation methods, which are limited in accuracy among spontaneously breathing patients. Therefore, treatment decisions were informed by the full HPI hemodynamic relations screen considering trend monitoring of SV and CI. If a contractility or afterload issue was identified, ephedrine 10 mg intravenous push was given. At the second HPI alert, the same approach was followed. At the third HPI alert, treatment was per physician anesthesiologist of record.

### 2.7. Self-Reported and Medical Record Data

Subjects were asked to report pain score immediately prior to ELA initiation, nausea, vomiting, or lightheadedness at any time during the monitoring period.

Data abstracted from the medical record included age, race, education level, smoking status, gravidity, parity, estimated gestational age, body mass index, induction vs. spontaneous labor, last known cervical exam at the time of ELA initiation, pain score immediately prior to ELA initiation, total vasopressor doses from time of ELA initiation until delivery, changes in fetal heart rate category, presence or absence of fetal heart rate decelerations within 1 h of initiation of ELA, mode of delivery, neonatal weight, neonatal Apgar scores, and neonatal cord blood pH.

*Study Efficacy Endpoints.* The primary endpoint was time-to-treatment of hypotension. Time-to-treatment was chosen as the primary endpoint because earlier treatment is hypothesized to mitigate downstream hemodynamic instability and improve maternal–fetal outcomes. Although less conventional than area under the curve (AUC) MAP < 65 mmHg or time-weighted averages, it offers an implementation-relevant measure of system responsiveness and clinical workflow efficiency in a labor unit setting.

Secondary endpoints were total minutes in hypotension accumulated from any time intervals with MAP < 65 mmHg based on ClearSight™ readings; nausea; vomiting; total events of SBP drop > 20% from baseline based on NIBP; total events of SBP < 100 mmHg based on NIBP; changes in fetal heart rate category; presence or absence of fetal rate decelerations within 1 h of initiation of ELA; total vasopressor doses (i.e., phenylephrine in mcg and ephedrine in mg); total intravenous fluids in labor (mL); and hemodynamic variables (cardiac output, CO; cardiac index, CI; stroke volume, SV; stroke volume variability, SVV; and systemic vascular resistance, SVR).

### 2.8. Feasibility and Acceptability Assessment

At the end of the monitoring period, subjects and their bedside nurses completed the Acceptability of Intervention Measure (AIM), Intervention Appropriateness Measure (IAM), and Feasibility of Intervention Measure (FIM) instruments—validated, standardized 4-item implementation outcome measures that assess perceived acceptability, appropriateness, and feasibility on a 5-point Likert scale ranging from “completely disagree” to “completely agree” [16,17]. The AIM, IAM, and FIM are measures to determine the degree to which respondents believe the intervention is acceptable, appropriate, and feasible, and these are often used to assess the probability of implementation success. An average score ≥ 4 was considered a positive rating. This approach has been previously validated for evaluating interventions in clinical implementation research.

### 2.9. Statistical Analysis

*Sample Size and Power Justification.* The primary outcome was time-to-treatment of hypotension. All analyses were conducted using a per-protocol approach to evaluate the feasibility, acceptability, and preliminary efficacy of the intervention under ideal conditions, consistent with the pilot nature and implementation focus of the study. Only participants who completed the full monitoring protocol and who had complete outcome data were included in the analysis. Based on a meta-analysis of trials, the estimated incidence of hypotension after initiation of neuraxial analgesia during labor is approximately 19% (range: 3–35%) [15], depending on population, definition, and timing of measurements. Cox proportional hazard models were used for this trial involving time-to-event analysis [18]. For a fully powered study, a sample of 74 patients (37 in each intervention group) experiencing hypotension will show a hazard ratio of 0.50 with power of 80% and two-sided α = 0.05, and a total of n = 370 would need to be enrolled with 20% (n = 74) experiencing hypotension events of interest. For this initial phase, we enrolled a target sample of 30 participants to evaluate study workflow, data completeness, subject and clinician acceptability, and feasibility and to explore treatment effect estimates that can inform the design and planning of a larger definitive trial. This sample size was not intended to power formal hypothesis testing on time-to-treatment but rather was sufficient to assess real-world feasibility, acceptability, and preliminary efficacy signals under the study protocol. The efficacy analyses presented in this study provide estimates of effect directionality and variability, both of which are critical for determining the practicality and design requirements of subsequent studies.

*Summary Statistics and Endpoint Comparisons by Monitoring Groups.* For continuous data, mean with standard deviation and median with interquartile range were both reported. For categorical variables, number with percentage was reported. Baseline characteristics and study endpoints were compared between treatment groups using nonparametric Kruskal–Wallis test for continuous variables and chi-square test or Fisher’s exact test for categorical variables.

*Time-to-Event Analysis for Primary Endpoint.* The Kaplan–Meier survival curves estimated the cumulative probability of time to first hypotension treatment over 4 h of monitoring period between Group CM and Group HPI. Log-rank test was used to compare if the entire Kaplan–Meier survival curves differs by groups. Proportional hazards regression models were used to estimate hazard ratios and 95% confidence intervals (CI) to assess the effect of monitoring type on time to hypotension treatment.

*Hemodynamic Outcomes*. Time series data for each hemodynamic outcome were averaged at every 15 min interval. Mean and bootstrap 95% CI were used to illustrate the trends and differences between the two groups for CO, CI, SV, SVV, and SVR at each 15 min time interval. Random intercept mixed-effects models were used to estimate if each hemodynamic outcome differs between monitoring groups during the entire 4 h monitoring period accounting for categorical time intervals. Interaction terms between monitoring groups and categorical time intervals were added to test if the change of hemodynamic outcomes over time differs by groups.

All statistical analyses were conducted using SAS version 9.4 (SAS Institute, Cary, NC) and R version 4.1.2 (R Foundation for Statistical Computing, Vienna, Austria). A *p* < 0.05 was used to define statistical significance.

## 3. Results

### 3.1. Study Population and Characteristics

**Figure 1:**
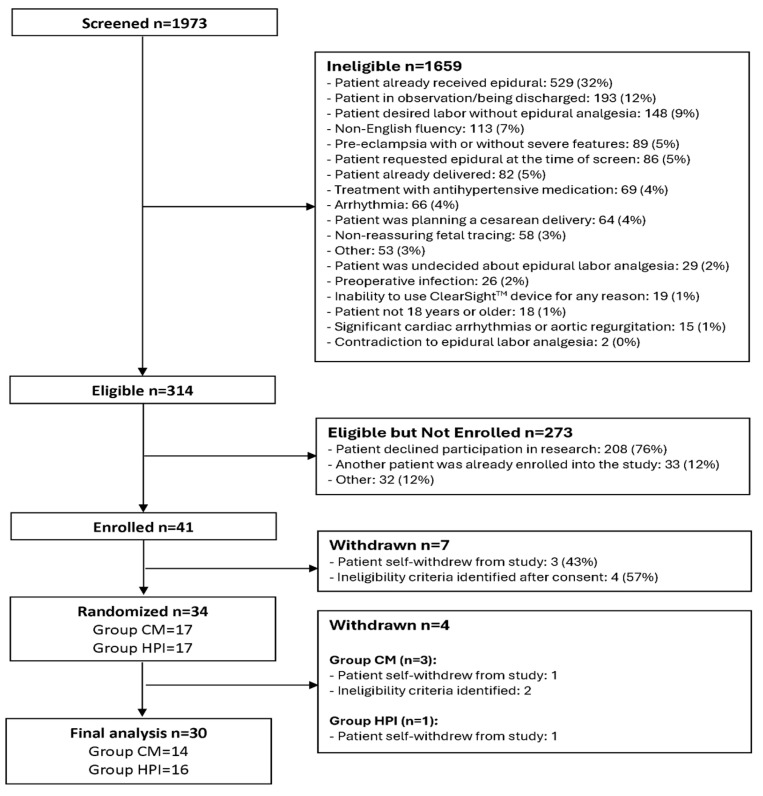
CONSORT diagram. CONSORT, consolidated standards of reporting trials.

A total of 314 eligible participants were identified, and 41 (13%) were enrolled with signed informed consent forms, while 7 were withdrawn before randomization. Among 34 randomized participants (17 in Group CM and 17 in Group HPI), 3 additional participants were withdrawn from Group CM, and 1 additional participant was withdrawn from Group HPI. There were 30 subjects with complete data included in the final analysis (Figure 1) with 14 (47%) randomized to Group CM and 16 (53%) to Group HPI. Mean (SD) age was 29.6 (5.5) years, and 83% were White, 13% were Black, and 3% were Asian. Mean (SD) of BMI was 33.7 (5.8) kg/m2, and pain before epidural was 5.6 (3.1). There were no statistically significant differences between Group CM and HPI for baseline and self-reported characteristics (Table 1).

### 3.2. Feasibility, Appropriateness, and Acceptability

**Figure 2:**
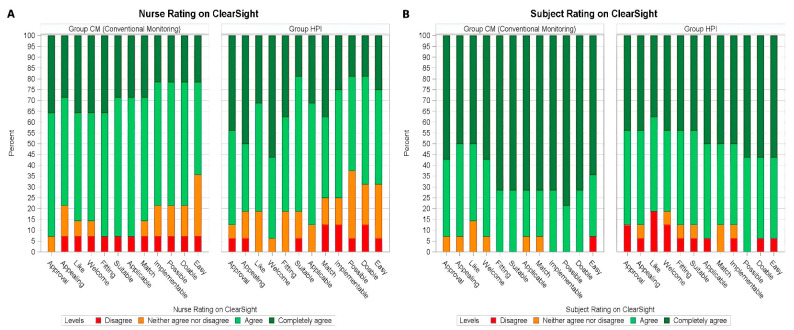
Heat map for subjects and nurse qualitative ratings on device acceptability. Ratings were given for the following 11 questions: The device meets my approval; The device is appealing to me; I like the device; I welcome the device; The device seems fitting; The device seems suitable; The device seems applicable; The device seems like a good match; The device seems implementable; The device seems possible; The device seems doable. Ratings were given on a 5-point Likert scale ranging from completely disagree to completely agree with these statements. (**A**) Subject ratings. (**B**) Nurse ratings.

Both patient and nurse ratings for AIM, IAM, and FIM were primarily positive, indicating that both groups rated the study device with high acceptability, appropriateness, and feasibility. Both patients and nurses rated the intervention highly across all implementation domains (median score ≥ 4 for all items) (Figure 2).

### 3.3. Primary Endpoint: Time to First Hypotension Treatment

The frequency of any hypotensive events was not different between Group CM (71%) and Group HPI (63%). Among 20 subjects who experienced hypotensive events, the median time-to-treatment was 14.5 min in Group CM and 16.0 min in Group HPI (Table 2). The Kaplan–Meier survival analysis estimated the first hypotension treatment probability for patients in Group CM and Group HPI. At 75 min, the cumulative probability for Group CM was 71%, compared to 65% for Group HPI, and remained the same probabilities through the end of the 4 h monitoring period. The survival difference was not statistically significantly different (log-rank *p* = 0.66), indicating that patients in Group CM and Group HPI were similar with respect to the probability of time to first hypotension treatment (Supplemental Figure S1). The Cox proportional hazards regression model shows an HR of 0.82 (95% CI: 0.34–1.98) in Group HPI compared to Group CM (Supplemental Table S1).

### 3.4. Secondary Analysis—Clinical and Hemodynamic Outcomes

Total minutes with MAP < 65 based on CBPM were similar between the groups (median 3.0 min in Group CM vs. 4.7 min in Group HPI; *p* > 0.99). There were trends toward higher rates of vomiting in Group CM (21%) compared to Group HPI (0%) (*p* = 0.09) (Table 2). There were no differences between Group CM and Group HPI for NIBP-measured frequency of SBP drop > 20% from baseline, SBP <100 mmHg, and fetal heart rate tracing concerns (Table 2). Among 20 subjects who experienced hypotensive events, total doses of ephedrine and phenylephrine and total intravenous fluid volumes were not different between groups (Table 2).

**Figure 3:**
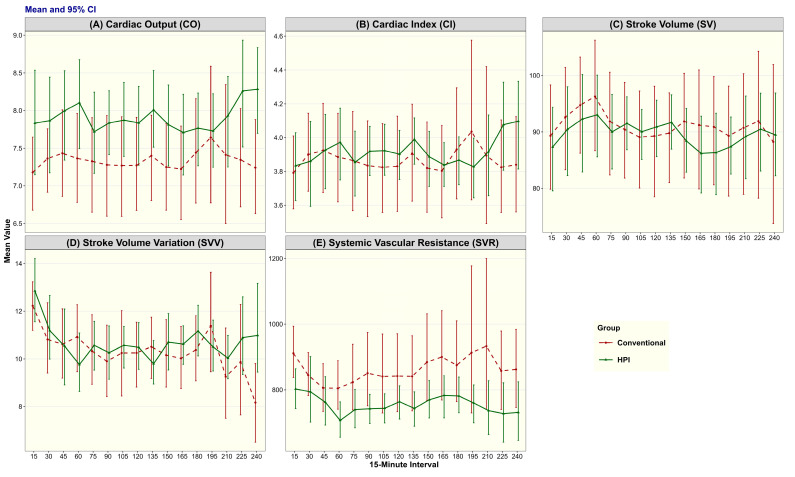
Mean and bootstrap 95% confidence interval of hemodynamic outcomes over time in 15 min intervals by treatment groups. Trends indicated differences between groups during the monitoring period for all hemodynamic variables measured but potentially were most beneficial for cardiac output, cardiac index, and systemic vascular resistance. (**A**) Cardiac output, CO. (**B**) Cardiac index, CI. (**C**) Stroke volume, CV. (**D**) Stroke volume variability, SVV. (**E**) Systemic vascular resistance, SVR.

CO was higher for Group HPI over the entire monitoring period (β = 0.58; 95% CI: −0.18, 1.34, *p* = 0.13). High SVR is a physiological risk factor known to influence placental and fetal perfusion. SVR was lower in Group HPI for the entire monitoring period (β = −97.22; 95% CI: −200.84, 6.40, *p* = 0.07), even though it was not statistically significant. The rate of change over time in hemodynamic variables was not significantly different by monitoring groups after testing the interaction term between group and time variables in random intercept mixed-effects models. (Table S2, Figure 3).

## 4. Discussion

The primary findings of this feasibility trial are that an HPI-guided hypotension clinical trial protocol in labor and delivery is feasible, appropriate, and acceptable to patients and clinicians and that treatment according to HPI may improve maternal hemodynamics (i.e., cardiac output, cardiac index, and systemic vascular resistance) during labor and delivery. These findings are significant because they suggest that real-time hemodynamic monitoring using HPI has the potential to lead to reduced risks associated with delayed treatment (e.g., compromised maternal hemodynamics and maternal–fetal perfusion). Our results provide further justification for future studies using larger sample sizes to make definitive conclusions about the potential role of HPI-guided therapies for modern epidural labor analgesia hypotension management. These findings are significant because they suggest that real-time hemodynamic monitoring using HPI is not only technically feasible but may be practical for scalable clinical integration into routine labor care.

Previous studies have shown comparable results favoring benefits of HPI-guided therapies. In one randomized trial [19], HPI was used with goal-directed therapy to manage intraoperative hypotension. The study reported a reduction in the number of hypotensive episodes when using HPI, particularly for optimization of hemodynamics, fluid, and vasopressor administration. Our study considered the findings from Maheshwari et al. [13], which suggested that lower alert thresholds and simpler treatment protocols would improve outcomes. Our HPI treatment index was set below that of Maheshwari et al., and our protocol was considerate of treatment norms in our hospital. Our findings support that a lower HPI trigger and a simple treatment protocol have the potential to improve maternal hemodynamics and are feasible and acceptable to subjects and clinicians alike. Although these results support that this approach remains theoretically beneficial for placental perfusion, this study was not designed to evaluate improvements in perfusion outcomes.

To our knowledge, this is the first study assessing HPI-guided therapies specifically in labor and delivery for the purpose of improving outcomes associated with epidural labor analgesia. Previous studies have primarily focused on non-obstetric populations, such as intraoperative hypotension management in non-cardiac surgery, where HPI-guided therapy has had variable results on hypotension outcomes and markers of tissue perfusion [13,20]. Research in obstetric-specific contexts is necessary to optimize protocols for HPI in childbirth. Our findings show smaller confidence intervals in Group HPI around several hemodynamic parameters, which suggests that HPI-guided treatment for ELA-associated hypotension may result in more precise control of hemodynamic targets. These findings may be primarily because HPI-guided treatment enables more precise therapies according to specific hemodynamic perturbations (e.g., correcting a systemic vascular resistance issue with vasopressors, correcting a preload issue with volume). Targeted hemodynamic therapies have implications for clinically relevant outcomes in the birth context, specifically for improving placental perfusion and prevention of emergent interventions for fetal distress that may result from placental insufficiency during labor and delivery. Figure 2B reveals subtle differences in nurse-reported feasibility between groups, with 1–2 responses indicating neutral or disagreement. This variation highlights potential device usability preferences and should be monitored in future investigations. Nevertheless, average scores across all domains met or exceeded the threshold of ≥ 4 on the 5-point Likert scale, indicating high perceived feasibility, appropriateness, and acceptability and thereby validating the primary feasibility aim.

We were surprised to see that our study shows 63–71% for hypotensive events, which is higher than previous estimates. Notably, in published literature, the quoted incidence of hypotension after epidural labor analgesia is highly variable, with estimates ranging between 14% and 36% [15,21,22]. These variations can be attributed to differences in study populations, definitions of hypotension, and clinical practices. In our study, we used a definition of hypotension that was consistent with our existing hospital policies and the definition of hypotension that informed HPI technology. Some explanations for our measured differences compared to what has been previously quoted may be because our institutional definitions of hypotension are more conservative than published literature, thereby leading to more patients meeting the hypotension definition, or because patients who elected to participate were interested because they were more prone to hypotensive events: that is, it is possible that individuals who consented to participate had prior experiences or concerns about hypotension that made them more inclined to join a monitoring study, although this hypothesis is speculative. We did not observe statistically significant differences in vasopressor or fluid usage between groups. This observation likely reflects the study’s small sample size and early-phase design. These measures should be examined in adequately powered trials designed to detect treatment effect magnitude. Symptoms such as nausea, vomiting, and fetal heart rate changes may result from a combination of factors, including labor progression, medications, and positioning, in addition to hypotension. Our study was not designed to delineate these etiologies. Fetal heart rate deceleration rates in our study were similar between CM and HPI groups, and all events were transient and clinically benign without requirement for any urgent interventions. Larger studies are needed to correlate specific hemodynamic changes with fetal monitoring outcomes. Despite trending differences, the lack of statistical significance in time-to-treatment likely reflects our feasibility sample size.

This study has several limitations. First, the sample size of 30 participants was intentionally small as a feasibility study. The small sample limits the making of definitive conclusions, but it was helpful in indicating trends to inform future trials. In our study, hemodynamic trends indicate potential benefits of HPI-guided therapies, particularly for cardiac output and systemic vascular resistance. A larger trial is needed to validate these results across more diverse populations. Second, the study duration was limited to 4 h of monitoring following epidural labor analgesia (ELA), which may not capture late-onset hypotension events or other long-term hemodynamic changes. Additionally, differences in monitoring frequency between groups—CBPM providing continuous data vs. NIBP cycling every 3–15 min—may have influenced hypotension detection and treatment response. This discrepancy reflects real-world differences between intermittent and continuous monitoring and underscores the need to interpret incidence comparisons with caution. Future trials can expand the monitoring period. Additionally, the study relied on continuous monitoring from the CBPM system, and while this technology has shown promise, the comparison with intermittent non-invasive blood pressure (NIBP) monitoring may not fully reflect real-world clinical conditions where frequent interruptions or device malfunctions could occur. Our population consisted of healthy term parturients with few comorbidities, limiting generalizability to high-risk populations where hypotension-related complications may be clinically significant. Furthermore, although we used standard spinal dosing for CSE analgesia, we acknowledge that lower dosing strategies may affect hypotension incidence—although they may also provide inferior analgesia—and therefore spinal dosing considerations may merit exploration in future studies.

A significant proportion of eligible patients (76%) declined participation, a trend commonly seen in labor studies requiring additional monitoring or consent under dynamic conditions. Primary reasons included discomfort with added monitoring, desire to minimize interventions, or declining research participation during active labor. These findings emphasize recruitment barriers in labor and delivery research and should be factored into planning for future larger-scale studies.

This study did not include area under the curve (AUC) MAP < 65 mmHg or time-weighted hypotension metrics, which are commonly used in intraoperative hypotension research. However, the clinical setting and objectives of this study differed from conventional studies: our focus was on feasibility, acceptability, and care responsiveness in a dynamic labor environment, where time-to-treatment serves as a more actionable and patient-centered implementation outcome. These conventional efficacy endpoints will be considered for incorporation into an analytic plan for a larger and fully powered trial on efficacy.

Our results suggest that adopting a protocol focused on earlier treatment of hypotension in labor and delivery can result in improvements in maternal physiology and symptoms. These improvements may also translate to improved fetal–neonatal outcomes, although trials are needed to assess the impact of HPI-driven protocols on fetal–neonatal outcomes specifically.

Future studies should explore long-term maternal and neonatal outcomes associated with HPI-guided interventions, including outcomes for the entirety of labor and delivery and postpartum. Larger trials in diverse populations would be valuable to validate the effectiveness of HPI across patient groups and settings and to optimize the alert thresholds and treatment algorithms. Also, examining the cost-effectiveness of implementing continuous HPI monitoring on labor units will be critical in determining its value and practicality in routine clinical practice across all practice settings. Research should also assess the impact of HPI-guided management on preventing adverse fetal outcomes that are attributable to maternal hypotension.

## 5. Conclusions

In conclusion, this study provides evidence that continuous monitoring and HPI-driven protocols to detect and treat hypotension in labor and delivery are feasible, appropriate, and acceptable to patients and clinicians. Although this study was not designed or intended to be powered to detect significant differences in time-to-treatment, we detected interesting trends in higher cardiac output and lower systemic vascular resistance, suggesting the potential for clinical benefit with HPI-guided treatment. These findings support further exploration in a larger trial.

HPI-guided treatment requires further definitive studies, as it represents a promising digital strategy to improve maternal hemodynamics and exemplifies how AI-driven monitoring can advance precision and responsiveness in obstetric anesthesia care. It has the potential to support earlier detection and precision treatment of epidural-associated hypotension, a common childbirth event with maternal and fetal safety implications. Future trials are needed to fully inform the clinical utility of HPI-guided labor anesthetic management. Future work should also explore how continuous monitoring and prediction platforms can be integrated with electronic medical records and clinical decision support systems to streamline implementation and ensure timely clinical response.

## Figures and Tables

**Table 1:** Patient characteristics of the study cohort.

Variable	All n = 30	Group CM n = 14 (47%)	Group HPI n = 16 (53%)	*p*-Value
**Age (years)**				
Mean (SD)	29.6 (5.5)	29.4 (5.5)	29.8 (5.7)	0.82
Median (25th:75th)	30.5 (26.0:34.0)	29.5 (24.0:35.0)	31.0 (26.0:33.5)	
**Race**				
White	25 (83%)	12 (86%)	13 (81%)	
Black	4 (13%)	1 (7%)	3 (19%)	0.60
Asian	1 (3%)	1 (7%)	0 (0%)	
**Education**				
High school or equivalent	2 (7%)	0 (0%)	2 (13%)	
Some college or technical training	6 (20%)	4 (29%)	2 (13%)	
Bachelor’s or associate’s degree	14 (47%)	5 (36%)	9 (56%)	0.06
Master’s degree	4 (13%)	1 (7%)	3 (19%)	
Doctorate or professional degree	4 (13%)	4 (29%)	0 (0%)	
**Smoking Status**				
Never smoker	20 (67%)	10 (71%)	10 (63%)	
Current smoker	4 (13%)	1 (7%)	3 (19%)	0.75
Former smoker	6 (20%)	3 (21%)	3 (19%)	
**BMI (kg/m^2^)**				
Mean (SD)	33.7 (5.8)	32.4 (5.1)	34.9 (6.3)	0.30
Median (25th:75th)	33.6 (28.9:37.2)	32.7 (27.6:35.1)	34.3 (33.3:37.4)	
**Gravidity**				
1	11 (37%)	5 (36%)	6 (38%)	
2	8 (27%)	4 (29%)	4 (25%)	0.99
3+	11 (37%)	5 (36%)	6 (38%)	
**Parity**				
0	13 (43%)	6 (43%)	7 (44%)	
1	10 (33%)	5 (36%)	5 (31%)	0.99
2	7 (23%)	3 (21%)	4 (25%)	
**Estimated Gestational Age (Weeks)**				
Mean (SD)	39.2 (1.2)	39.2 (1.0)	39.2 (1.4)	
Median (25th:75th)	39.1 (38.7:40.1)	39.1 (38.3:39.7)	39.2 (38.9:40.2)	0.12
Min:Max	35.9:41.1	37.6:41.1	35.9:41.0	
**Labor Method**				
Induction	21 (70%)	12 (86%)	9 (56%)	0.12
Spontaneous labor	9 (30%)	2 (14%)	7 (44%)	
**Pain Score Before ELA** **(0–10 Numeric Rating Scale)**				
Mean (SD)	5.6 (3.1)	5.4 (2.6)	5.8 (3.5)	0.40
Median (25th:75th)	6.5 (4.0:8.0)	6.0 (4.0:7.0)	7.5 (2.5:8.5)	

CM, conventional monitoring. HPI, Hypotension Prediction Index. SD, standard deviation. BMI, body mass index. ELA, epidural labor analgesia.

**Table 2:** Clinical outcomes among all subjects and outcomes among subjects with hypotensive events.

***All Subjects***				
**Variable**	**All Subjects** **n = 30**	**Group CM** **n = 14**	**Group HPI** **n = 16**	***p*-value**
**Hypotensive Event (Any)**	**n = 30**	**n = 14**	**n = 16**	**0.70**
No	10 (33%)	4 (29%)	6 (38%)	
Yes	20 (67%)	10 (71%)	10 (63%)	
**Hypotensive Treatment (Any)**	**n = 30**	**n = 14**	**n = 16**	**0.70**
No	10 (33%)	4 (29%)	6 (38%)	
Yes	20 (67%)	10 (71%)	10 (63%)	
**Total Number of Hypotensive Events**	**n = 30**	**n = 14**	**n = 16**	**0.49**
Mean (SD)	2.0 (2.0)	2.2 (1.9)	1.8 (2.0)	
Median (25th:75th)	1.5 (0.0:3.0)	2.5 (0.0:4.0)	1.0 (0.0:3.0)	
Min:Max	0.0:7.0	0.0:6.0	0.0:7.0	
**MAP< 65 Total Minutes Based on CBPM**	**n = 30**	**n = 14**	**n = 16**	**0.99**
Mean (SD)	10.0 (14.9)	12.5 (19.3)	7.7 (9.5)	
Median (25th:75th)	3.4 (0.7:19.3)	3.0 (0.7:25.3)	4.7 (0.7:13.3)	
Min:Max	0.0:62.3	0.0:62.3	0.0:30.3	
**Total Events of SBP drop > 20% from Baseline Based on NIBP**	**n = 30**	**n = 14**	**n = 16**	**0.61**
Mean (SD)	3.6 (3.8)	3.4 (3.6)	3.8 (4.1)	
Median (25th:75th)	2.0 (1.0:6.0)	1.5 (0.0:7.0)	2.5 (1.0:5.0)	
**Total Events of SBP < 100 mmHg Based on NIBP**	**n = 30**	**n = 14**	**n = 16**	**0.37**
Mean (SD)	2.9 (2.8)	3.1 (2.5)	2.8 (3.1)	
Median (25th:75th)	2.0 (1.0:4.0)	3.0 (2.0:5.0)	2.0 (1.0:3.5)	
**Nausea (Any)**	**n = 29**	**n = 14**	**n = 15**	**0.71**
No	17 (59%)	9 (64%)	8 (53%)	
Yes	12 (41%)	5 (36%)	7 (47%)	
**Vomiting (Any)**	**n = 30**	**n = 14**	**n = 16**	**0.09**
No	27 (90%)	11 (79%)	16 (100%)	
Yes	3 (10%)	3 (21%)	0 (0%)	
**Lightheadedness (Any)**	**n = 30**	**n = 14**	**n = 16**	**0.61**
No	27 (90%)	12 (86%)	15 (94%)	
Yes	3 (10%)	2 (14%)	1 (6%)	
**Changes in Fetal Heart Rate Category (Any)**	**n = 29**	**n = 14**	**n = 15**	**0.46**
No	15 (52%)	6 (43%)	9 (60%)	
Yes	14 (48%)	8 (57%)	6 (40%)	
**Fetal Heart Rate Decelerations within 1 h of Initiation of ELA**	**n = 29**	**n = 14**	**n = 15**	**0.99**
No	19 (66%)	9 (64%)	10 (67%)	
Yes	10 (34%)	5 (36%)	5 (33%)	
***Subjects with Hypotensive Events***				
**Variable**	**Subjects with** **Hypotensive Event n = 20**	**Group CM** **n = 10**	**Group HPI** **n = 10**	***p*-value**
**Time to First Hypotension Treatment (Min)**	**n = 20**	**n = 10**	**n = 10**	**0.88**
Mean (SD)	22.5 (17.1)	21.8 (17.5)	23.1 (17.6)	
Median (25th:75th)	14.5 (10.0:30.0)	14.5 (10.0:30.0)	16.0 (10.0:30.0)	
**Total Ephedrine (mg)**	**n = 19**	**n = 10**	**n = 9**	**0.42**
Mean (SD)	26.7 (24.6)	29.8 (26.4)	23.3 (23.6)	
Median (25th:75th)	20.0 (10.0:20.0)	20.0 (15.0:25.0)	20.0 (10.0:20.0)	
**Total Phenylephrine (mcg)**	**n = 9**	**n = 6**	**n = 3**	**0.15**
Mean (SD)	317.8 (314.4)	223.3 (242.1)	506.7 (410.5)	
Median (25th:75th)	160.0 (80.0:400.0)	120.0 (80.0:240.0)	400.0 (160.0:960.0)	
**Total Intravenous Fluids (mL)**	**n = 16**	**n = 9**	**n = 7**	**0.59**
Mean (SD)	543.8 (137.7)	550.0 (169.6)	535.7 (94.5)	
Median (25th:75th)	500.0 (500.0:500.0)	500.0 (500.0:500.0)	500.0 (500.0:500.0)	

MAP, mean arterial pressure. ELA, epidural labor analgesia. NIBP, non-invasive blood pressure. Mg, milligrams. Mcg, micrograms. mL, milliliters. SD, standard deviation. Min, minimum. Max, maximum. CM, conventional monitoring, HPI, Hypotension Prediction Index.

## Data Availability

The data that support the findings of this study are not publicly available due to ethical and privacy restrictions involving protected health information of study participants. De-identified data may be made available from the corresponding author (G.L.) upon reasonable request and with appropriate institutional approvals.

## Supplementary Materials

The following supporting information can be downloaded at: https://www.mdpi.com/article/10.3390/jcm14145037/s1, Figure S1: Cumulative probability of first hypotension treatment by monitoring group; Table S1: Hazard ratio of hypotension treatment by monitoring group; Table S2: Hemodynamic variables over time by monitoring group.

## References

[1] (2016). Practice Guidelines for Obstetric Anesthesia: An Updated Report by the American Society of Anesthesiologists Task Force on Obstetric Anesthesia and the Society for Obstetric Anesthesia and Perinatology. Anesthesiology.

[2] Zheng X., Siddiqui Z., Anderson N., Chen C.M., Chatterji M., Smiley R., Landau R., Meng M.L. (2020). Check the Blood Pressure!: An Educational Tool for Anesthesiology Trainees Converting Epidural Labor Analgesia to Cesarean Delivery Anesthesia. A A Pract..

[3] Dyer R.A., Reed A.R., van Dyk D., Arcache M.J., Hodges O., Lombard C.J., Greenwood J., James M.F. (2009). Hemodynamic effects of ephedrine, phenylephrine, and the coadministration of phenylephrine with oxytocin during spinal anesthesia for elective cesarean delivery. Anesthesiology.

[4] Hatib F., Jian Z., Buddi S., Lee C., Settels J., Sibert K., Rinehart J., Cannesson M. (2018). Machine-learning Algorithm to Predict Hypotension Based on High-fidelity Arterial Pressure Waveform Analysis. Anesthesiology.

[5] Frassanito L., Giuri P.P., Vassalli F., Piersanti A., Longo A., Zanfini B.A., Catarci S., Fagotti A., Scambia G., Draisci G. (2022). Hypotension Prediction Index with non-invasive continuous arterial pressure waveforms (ClearSight): Clinical performance in Gynaecologic Oncologic Surgery. J. Clin. Monit. Comput..

[6] Frassanito L., Sonnino C., Piersanti A., Zanfini B.A., Catarci S., Giuri P.P., Scorzoni M., Gonnella G.L., Antonelli M., Draisci G. (2022). Performance of the Hypotension Prediction Index with Noninvasive Arterial Pressure Waveforms in Awake Cesarean Delivery Patients Under Spinal Anesthesia. Anesth. Analg..

[7] Juri T., Suehiro K., Kimura A., Mukai A., Tanaka K., Yamada T., Mori T., Nishikawa K. (2018). Impact of non-invasive continuous blood pressure monitoring on maternal hypotension during cesarean delivery: A randomized-controlled study. J. Anesth..

[8] Maheshwari K., Buddi S., Jian Z., Settels J., Shimada T., Cohen B., Sessler D.I., Hatib F. (2021). Performance of the Hypotension Prediction Index with non-invasive arterial pressure waveforms in non-cardiac surgical patients. J. Clin. Monit. Comput..

[9] Helmer P., Helf D., Sammeth M., Winkler B., Hottenrott S., Meybohm P., Kranke P. (2022). The Use of Non-Invasive Continuous Blood Pressure Measuring (ClearSight(^®^)) during Central Neuraxial Anaesthesia for Caesarean Section-A Retrospective Validation Study. J. Clin. Med..

[10] Davies S.J., Vistisen S.T., Jian Z., Hatib F., Scheeren T.W.L. (2020). Ability of an Arterial Waveform Analysis-Derived Hypotension Prediction Index to Predict Future Hypotensive Events in Surgical Patients. Anesth. Analg..

[11] Pinsky M.R., Cecconi M., Chew M.S., De Backer D., Douglas I., Edwards M., Hamzaoui O., Hernandez G., Martin G., Monnet X. (2022). Effective hemodynamic monitoring. Crit. Care.

[12] Sidiropoulou T., Tsoumpa M., Griva P., Galarioti V., Matsota P. (2022). Prediction and Prevention of Intraoperative Hypotension with the Hypotension Prediction Index: A Narrative Review. J. Clin. Med..

[13] Maheshwari K., Shimada T., Yang D., Khanna S., Cywinski J.B., Irefin S.A., Ayad S., Turan A., Ruetzler K., Qiu Y. (2020). Hypotension Prediction Index for Prevention of Hypotension during Moderate- to High-risk Noncardiac Surgery. Anesthesiology.

[14] Kinsella S.M., Black A.M. (1998). Reporting of ‘hypotension’ after epidural analgesia during labour. Effect of choice of arm and timing of baseline readings. Anaesthesia.

[15] Simmons S.W., Taghizadeh N., Dennis A.T., Hughes D., Cyna A.M. (2012). Combined spinal-epidural versus epidural analgesia in labour. Cochrane Database Syst. Rev..

[16] Proctor E., Silmere H., Raghavan R., Hovmand P., Aarons G., Bunger A., Griffey R., Hensley M. (2011). Outcomes for implementation research: Conceptual distinctions, measurement challenges, and research agenda. Adm. Policy Ment. Health.

[17] Weiner B.J., Lewis C.C., Stanick C., Powell B.J., Dorsey C.N., Clary A.S., Boynton M.H., Halko H. (2017). Psychometric assessment of three newly developed implementation outcome measures. Implement. Sci..

[18] Schoenfeld D.A. (1983). Sample-size formula for the proportional-hazards regression model. Biometrics.

[19] Šribar A., Jurinjak I.S., Almahariq H., Bandić I., Matošević J., Pejić J., Peršec J. (2023). Hypotension prediction index guided versus conventional goal directed therapy to reduce intraoperative hypotension during thoracic surgery: A randomized trial. BMC Anesthesiol..

[20] Lorente J.V., Ripollés-Melchor J., Jiménez I., Becerra A.I., Mojarro I., Fernández-Valdes-Bango P., Fuentes M.A., Moreno A., Agudelo M.E., Villar-Pellit de la Vega A. (2023). Intraoperative hemodynamic optimization using the hypotension prediction index vs. goal-directed hemodynamic therapy during elective major abdominal surgery: The Predict-H multicenter randomized controlled trial. Front. Anesthesiol..

[21] Ghidini A., Vanasche K., Cacace A., Cacace M., Fumagalli S., Locatelli A. (2024). Side effects from epidural analgesia in laboring women and risk of cesarean delivery. AJOG Glob. Rep..

[22] Korb D., Bonnin M., Michel J., Oury J.F., Sibony O. (2013). Analysis of fetal heart rate abnormalities occurring within one hour after laying of epidural analgesia. J. Gynecol. Obs. Biol. Reprod..

