# Supplementary material for: Feasibility of Hypotension Prediction Index-Guided Monitoring for Epidural Labor Analgesia: A Randomized Controlled Trial
Source: J Clin Med. 2025 Jul 16;14(14):5037. doi: 10.3390/jcm14145037 (PMC12295593; doi:10.3390/jcm14145037)
Supplement: Supplementary file 1 [file jcm-14-05037-s001.zip › jcm-3633276-supplementary.pdf]

SUPPLEMENTAL MATERIAL

Supplemental Figure S1. Cumulative probability of first hypotension treatment by monitoring group.

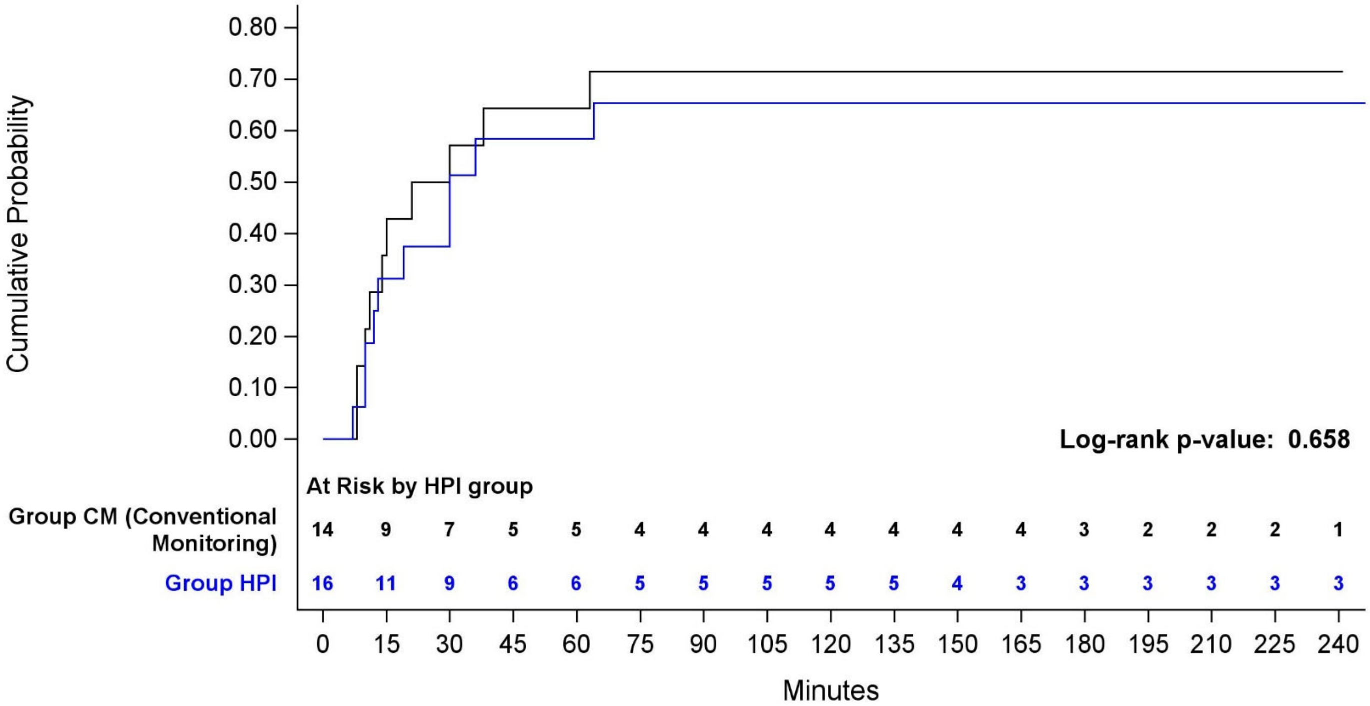

Cumulative Probability by HPI group

**Group CM (Conventional Monitoring)**	**0.43**	**0.57**	**0.64**	**0.64**	**0.71**	**0.71**	**0.71**	**0.71**	**0.71**	**0.71**	**0.71**	**0.71**	**0.71**	**0.71**	**0.71**	**0.71**
**Group HPI**	**0.31**	**0.51**	**0.58**	**0.58**	**0.65**	**0.65**	**0.65**	**0.65**	**0.65**	**0.65**	**0.65**	**0.65**	**0.65**	**0.65**	**0.65**	**0.65**

Supplemental Table S1. Hazard Ratio of Hypotension Treatment by Monitoring Group

Variable	Hazard Ratio	95% CI	*P*-value
**Unadjusted Model**			
Group HPI	0.82	0.34-1.98	0.66
Group CM	Reference	Reference	

Abbreviations: CM: conventional monitoring. HPI, hypotension prediction index. CI: confidence interval

Supplemental Table S2. Hemodynamic variables over time by monitoring group.

**Outcome**	**Effect***	**Beta Coefficients (95% CI)**	***P*-value**
Cardiac Output (CO)	Group HPI	0.58 (-0.18, 1.34)	0.13
Cardiac Index (CI)	Group HPI	0.03 (-0.26, 0.32)	0.86
Stroke Volume (SV)	Group HPI	-0.35 (-10.86, 10.17)	0.95
Stroke Volume Variability (SVV)	Group HPI	0.19 (-1.28, 1.65)	0.80
Systemic Vascular Resistance (SVR)	Group HPI	-97.22 (-200.84, 6.40)	0.07

*Group HPI vs Group CM as the reference group.

Random intercept mixed-effects models were used to estimate if each hemodynamic outcome differs between monitoring groups over time in 15-min intervals. Interaction terms between monitoring groups and time were added to test if the change of hemodynamic variables differs by groups but none was significant.
